# Diversity and natural infection of phlebotomine sand flies (Diptera, Psychodidae) in an endemic area of American tegumentary leishmaniasis in southeastern Bahia, Brazil

**DOI:** 10.1186/s13071-025-06717-y

**Published:** 2025-02-26

**Authors:** Bruno Oliveira Cova, Livia Alves de Oliveira, Paulo Roberto Lima Machado, Edgar Marcelino de Carvalho, Adriano Figueiredo Monte-Alegre, Albert Schriefer

**Affiliations:** 1 Serviço de Imunologia, Hospital Universitário Professor Edgard Santos, Universidade Federal da Bahia (UFBA), Salvador, Brazil; 2 Departamento de Ciências da Biointeração, Instituto de Ciências da Saúde, UFBA, Salvador, Brazil; 3 Programa de Pós-Graduação Em Ciências da Saúde, Faculdade de Medicina da Bahia, UFBA, Salvador, Brazil; 4 Instituto Nacional de Ciência e Tecnologia em Doenças Tropicais (INCT-DT), Conselho Nacional de Desenvolvimento Científico e Tecnológico (CNPq), Salvador, Bahia Brazil; 5 Immunology Service of the Professor Edgard Santos Hospital Complex (COM-HUPES), Federal University of Bahia (UFBA), Augusto Viana Street, Canela, Salvador, Bahia Brazil

**Keywords:** American tegumentary leishmaniasis, Phlebotominae, *Leishmania* (*Viannia*) *braziliensis*, Bahia, Brazil

## Abstract

**Background:**

The Cacao Region spans several municipalities in the state of Bahia. It is one of the major foci of American tegumentary leishmaniasis (ATL) in Brazil. We report the findings of a pilot cross-sectional study describing the phlebotomine fauna found around living sites of newly diagnosed ATL cases in that area.

**Methods:**

The sand fly fauna was studied from May 2018 to June 2019 via an entomological survey, as recommended by the Brazilian Ministry of Health.

**Results:**

Six hundred nineteen phlebotomine sand flies of 20 species were captured: 272 males (44%) and 347 females (56%). *Nyssomyia whitmani* was the most prevalent (62.2%), followed by *Nyssomyia intermedia* (9.2%), *Evandromyia bahiensis* (6.3%), endemic to Bahia, and *Trichophoromyia viannamartinsi* (4.5%). Ninety-four percent of the female sand flies collected were screened for infection with *Leishmania* (*Viannia*) *braziliensis* by polymerase chain reaction (PCR). Of the 97 sand fly pools analyzed, seven were positive for *L.* (*V.*) *braziliensis*: three of *Nyssomyia whitmani*, two of *Th. viannamartinsi* and one each of *Psychodopygus hirsutus hirsutus* and *Trichopygomyia longispina*. The overall value of minimum infection rate (MIR) was 2.2%, and its stratification rates for the above species were 1.9, 10, 33 and 50%, respectively. All positive pools consisted of phlebotomine sand flies collected from the peridomiciles and extradomiciles of homes in the municipality of Taperoá in July 2018, resulting in an adjusted MIR of 7.8%, with 50% of the pools positive for *L.* (*V.*) *braziliensis* during that outbreak of ATL.

**Conclusions:**

Our findings suggest that areas experiencing outbreaks of ATL in affected regions present high proportions of infected phlebotomine sand flies involving a variety of species, some not usually considered involved in the *L.* (*V.*) *braziliensis* transmission cycle, such as *Th. viannamartinsi*.

**Graphical Abstract:**

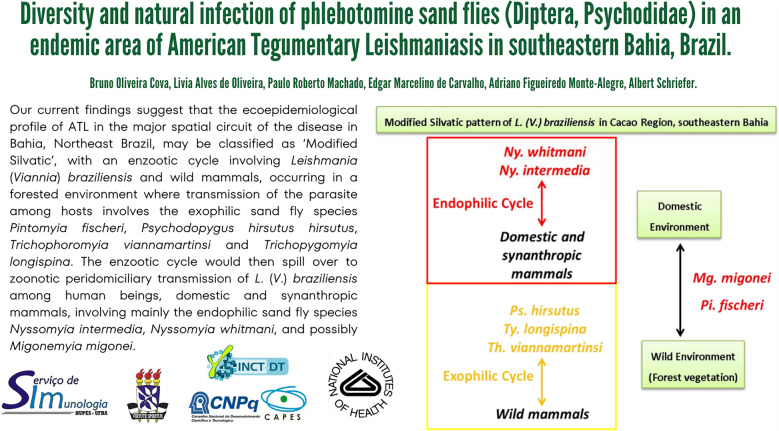

**Supplementary Information:**

The online version contains supplementary material available at 10.1186/s13071-025-06717-y.

## Background

American tegumentary leishmaniasis (ATL) is a neglected tropical disease, and Brazil is a country with a large number of reported human cases [1, 2]. ATL is characterized by single or multiple skin lesions and is undergoing territorial expansion, revealing changes in its epidemiological profile with an increase in the domestic transmission [3].

*Leishmania* (*Viannia*) *braziliensis* Vianna, 1911, is one of the species responsible for ATL cases and may occur in the form of localized cutaneous leishmaniasis (CL) and mucosal leishmaniasis (ML) [4]. This species has also been implicated in the emergence of disseminated cutaneous leishmaniasis (DL), in which patients may present with dozens to hundreds of skin lesions spread throughout different body parts, often involving the oropharyngeal mucosa. ML and DL are severe, hard-to-treat variants of ATL, which may result in disfiguring outcomes [5–8].

Complex transmission cycles involving a variety of vectors, hosts and reservoirs are involved in maintaining ATL-causative agents in nature [9]. The impact of natural or human-made ecological pressures can further lead to the establishment of new transmission cycles [10].

Phlebotomine sand flies (Diptera: Psychodidae) have great medical importance since their females can transmit leishmania parasites (*Leishmania* Ross, 1903; Euglenozoa: Trypanosomatidae: Leishmaniinae) [11]. Approximately 1000 different sand fly species have been described worldwide, 530 of which inhabit the Americas. At least 280 such species have been identified in Brazil and 76 in the Bahia state in the northeast of country. Thirty-nine species of phlebotomine sand flies are proven or putative vectors of ATL in Brazil, 27 in Bahia [9, 12–15].

The Brazilian public health authorities registered 300,000 ATL cases between 2003 and 2018. This figure results in an average of 21.158 cases/year and an incidence of 11.3 cases/100.000 inhabitants. ML accounted for 7.7% of the cases registered [16]. Bahia was the state most affected by ATL in northeast Brazil, ranking second nationwide, with approximately 37,000 notifications of the disease between 2007 and 2018 [17]. Human cases are chiefly distributed among four major spatial ATL circuits in Brazil: Vale do Jequitinhonha (Jequitinhonha valley), spanning the states of Minas Gerais and Bahia; Chapada Diamantina (Diamantina plateau), Coribe; and the Cacao Region in Bahia. The Cacao Region is located in southeastern Bahia, which is the main spatial ATL circuit in that state, with human cases reported since early Brazilian colonization [18].

Studies carried out in southeastern Bahia revealed dynamic spatiotemporal aggregation of ATL cases in this region [19, 20]. This suggests that the ideal conditions for the efficient transmission of the parasite to the human population and, therefore, for the maintenance of the endemic should be spatially concentrated during the transmission season of *L.* (*V.*) *braziliensis*.

Research on phlebotomine species diversity and relative abundance in natural foci of Leishmaniasis helps elucidate relevant aspects of the transmission cycle of *Leishmania* spp. to humans [21], such as its anthropophilic or zoophilic behavior, the effects of vegetation on the distribution of sand fly species and the identification of its resting and feeding locations [22].

Numerous sand fly species have been described as potential vectors of *L.* (*V.*) *braziliensis* [23]. However, the participation of at least some of these species in the effective establishment and maintenance of ATL endemics is still elusive. In the present study, we describe the phlebotomine fauna found around and within residences of newly diagnosed cases of ATL in the Cacao Region, southeastern Bahia, Brazil. We investigated the diversity and relative abundance of the sand fly species in different ecotypes relative to the living sites of ATL index cases as well as its *L.* (*V.*) *braziliensis* infection rates.

## Methods

### Study area

This descriptive cross-sectional study was carried out in a hyper-endemic focus on ATL caused by *L.* (*V.*) *braziliensis*, which is located in the Cacao Region spanning the Corte de Pedra district [7]. The Cacao Region comprises 20 municipalities within a rural area located in the southeast of the state of Bahia, northeast Brazil. It spans the following geographic coordinates (latitude/longitude): − 14°/− 39°, − 13°/− 39°, − 14°/− 40°, − 13°/− 40°. Residents of this area work mostly in agriculture, such as cacao, banana and clove crops, often carried out in primary or secondary forests.

### Patients and disease definitions

All included subjects resided in the *L.* (*V.*) *braziliensis* endemic region and were self-referred to and diagnosed at the leishmaniasis clinic in Corte de Pedra. Only patients presenting with localized cutaneous leishmaniasis (CL) or disseminated cutaneous leishmaniasis (DL) served as index cases in the study, although other less frequent ATL forms exist in the region. The clinical criteria for CL included < 10 ulcerative skin lesions without evidence of mucosal involvement. DL was defined as a disease with > 10 acneiform, papular or ulcerative skin lesions spread over two or more body areas, with or without mucosal involvement [5–7]. In addition to other standard ATL diagnostic procedures, all the subjects had infection confirmed by parasite DNA detection in lesion biopsy samples via real-time PCR [24].

### Study sample

**Fig. 1:**
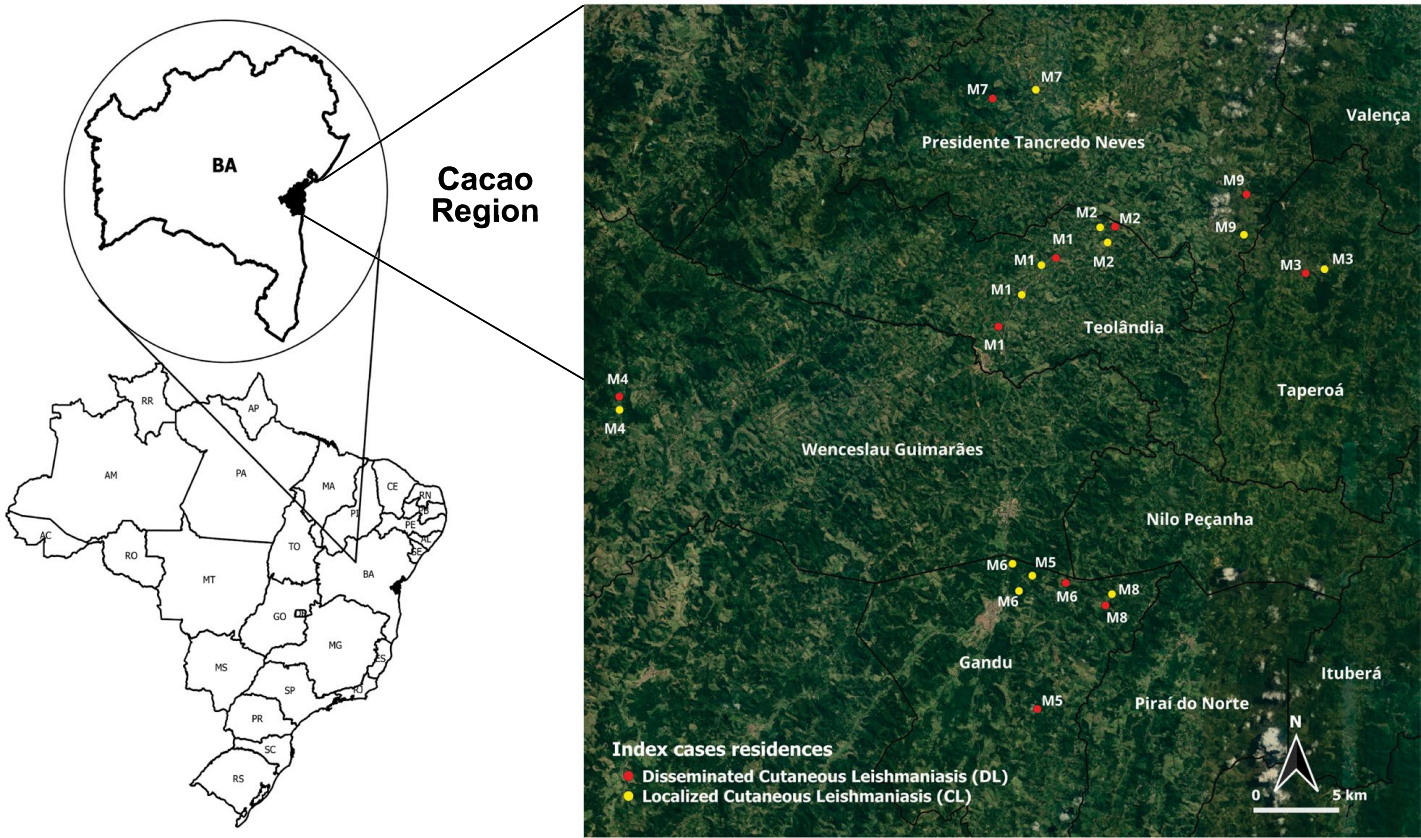
Phlebotomine sand fly collection sites in the Cacao Region, southern Bahia, Brazil. Entomological survey was performed as recommended by the Brazil Ministry of Health between 2018 and 2019. M1 = May 18; M2 = June 18; M3 = July 18; M4 = August 18; M5 = October 18; M6 = February 19; M7 = March 19; M8 = April 19; M9 = June 19

The study sample consisted of phlebotomine sand flies collected in the proximity of the residences of the CL and DL index cases, with lesions reported within the 60 days preceding the PCR-confirmed infection with *L.* (*V.*) *braziliensis*. The insects were captured in nine collections between May 2018 and June 2019, after 14 monthly visits to the endemic region. At least two geographically close index case residences were selected for sand fly collection in each visit: one from a patient with DL paired with one from a patient with CL (Fig. 1). The residences of these pairs were preferably located within 2.5 km distance to increase the chance that the two index cases participated in the same ongoing outbreak of ATL, in accordance with previous studies that described the dynamics of endemics in this region [19, 20].

The entomological surveys were performed as recommended by the Brazilian Ministry of Health [25]. Phlebotomine sand flies were collected monthly via Centers for Disease Control and Prevention (CDC) night-time light traps in the forest and in anthropic environments (peridomestic environment and inside human dwellings). We installed three CDC traps per residence at a height of 1 m for 12-h periods, from 6:00 p.m. to 6:00 a.m., two in the home environment (dormitories, external house walls and animal shelters) and one in the wild environment (extradomicile, forest edge up to 500 m from the residence).

We compared the phlebotomine fauna between geographically matched households presenting index cases of distinct ATL forms (i.e. CL or DL), analyzing significant differences in abundance, richness, diversity and endophily/exophily behaviors through nonparametric tests (Kruskal-Wallis and Mann-Whitney tests).

### Molecular detection and rate of phlebotomine sand fly infection with *L.* (*V.*)* braziliensis*

The thorax and first abdomen segment of each female sand fly were preserved for molecular analysis; the head and last abdominal segment were mounted between slides/coverslips, which were fixed in Hoyer’s solution for species identification. Taxonomic analyses were performed adopting Young & Duncan [26] and Galati [27], and the abbreviations of the sand fly genera are those proposed by Marcondes [28].

For the determination of infection rates with *L.* (*V.*) *braziliensis*, sand fly females were separated into pools of up to 10 individuals. The composition of each pool was determined by the date of collection, ecotype (intradomicile, peridomicile or extradomicile) and taxonomic identification.

DNA was extracted via the Invitrogen Genomic DNA Mini Kit^®^, following the manufacturer’s protocol. *Leishmania* (*V.*) *braziliensis* kDNA amplification from female sand flies’ DNA pools was performed via qPCR as previously described [24]. PCRs were carried out in a StepOnePlus^™^ Real-Time PCR System (Applied Biosystems^®^) via the TaqMan^®^ system. Positive pools were detected by interpolation into a standard curve of cultured *L.* (*V.*) *braziliensis* DNA amplified in parallel.

The *Leishmania* natural infection rates in the phlebotomine sand flies’ DNA pools were expressed as minimum infection rate (MIR), calculated as the ratio between the number of positive pools and the number of individuals within the tested pool × 100, as previously described [29]. The MIRs were calculated by species, ecotype and clinical form of ATL (i.e. CL or DL) presented by the index cases. We evaluated significant differences between positive pools of CL and DL living sites through Fisher’s nonparametric test.

## Results

From May 2018 to June 2019, 619 specimens of phlebotomine sand flies of 20 different species were captured at 20 positive collection points in the 22 homes surveyed: 272 males (44%) and 347 females (56%). The geographic distribution of the 22 phlebotomine sand fly collection sites spanned in five municipalities in the Cacao Region: Gandu, Presidente Tancredo Neves, Taperoá, Teolândia and Wenceslau Guimarães (Fig. 1).

The species *Ny. whitmani* was the most predominant at 62.2%, followed by *Nyssomyia intermedia* (9.2%), *Evandromyia bahiensis* (6.3%), which is endemic in Bahia [15], and *Th. viannamartinsi* (4.5%). The frequency distributions of all 20 species captured are displayed in Table 1. Fifteen percent of the individuals were collected in the intradomicile, 39% in the peridomicile and 46% in the local forest. Statistical significance was detected for the differences between the abundance of phlebotomine sand flies in these ecotypes (Kruskal-Wallis *H* test, *H* = 16.894, df = 2, *P* < 0.001). The intradomiciles had significantly lower abundances than the peridomiciles and extradomiciles of the residences surveyed (Mann-Whitney *U* test, *U* = 69.50, *Z* = − 3.12, *P* = 0.003; Mann-Whitney *U* test, *U* = 45.00, *Z* = − 3.87, *P* < 0.001, respectively).

**Table 1:** Phlebotomine sand fly species collected between May 2018 and June 2019 in the Cacao Region, Bahia, Brazil

Species	Intra		Peri		Extra		Total^a^		RA^a^
	♂	♀	♂	♀	♂	♀	♂	♀	
*Nyssomyia whitmani* (Antunes e Coutinho, 1939)	18	51	98	57	100	61	216 (56)	169 (44)	62.2
*Nyssomyia intermedia* (Lutz e Neiva, 1912)	03	14	08	16	03	13	14 (25)	43 (75)	9.2
*Evandromyia bahiensis* (Mangabeira e Sherlock, 1971)			02	13	03	21	05 (13)	34 (87)	6.3
*Trichophoromyia viannamartinsi* (Sherlock e Guitton, 1970)	02	03		07	05	11	07 (25)	21 (75)	4.5
*Pintomyia fischeri* (Pinto, 1926)		01	02	03	02	16	04 (17)	20 (83)	3.9
*Migonemyia migonei* (França, 1920)	02		05	08		06	07 (33)	14 (67)	3.4
*Micropygomyia schreiberi* (Martins, Falcão e Silva, 1975)		01		05	01	09	01 (6)	15 (94)	2.6
*Evandromyia tupynambai* (Mangabeira, 1942)		01	02	03	01	04	03 (27)	08 (73)	1.8
*Psathyromyia aragaoi* (Costa Lima, 1932)			02		03	05	05 (50)	05 (50)	1.6
*Pressatia choti* (Floch e Abonnenc, 1941)				02	02	05	02 (22)	07 (78)	1.5
*Psathyromyia bigeniculata* (Floch & Abonnenc, 1941)				01		02		03 (100)	0.5
*Psathyromyia pascalei* (Barretto e Coutinho, 1940)			02		01		03 (100)		0.5
*Psychodopygus hirsutus hirsutus* (Mangabeira, 1942)						03		03 (100)	0.5
*Micropygomyia oswaldoi* (Mangabeira, 1942)			01		01		02 (100)		0.3
*Pintomyia serrana* (Damasceno & Arouck, 1949)			01			01	01 (50)	01 (50)	0.3
*Trichopygomyia longispina* (Mangabeira, 1942)						02		02 (100)	0.3
*Brumptomyia cunhai* (Mangabeira, 1942)					01		01 (100)		0.2
*Micropygomyia capixaba* (Dias, Falcão, Silva e Martins, 1987)						01		01 (100)	0.2
*Psathyromyia barretoi barretoi* (Mangabeira, 1942)				01				01 (100)	0.2
*Psathyromyia lanei* (Barretto & Coutinho, 1941)			01				01 (100)		0.2
Total^a^	25	71	124	116	123	160	272 (44)	347 (56)	100
	96 (15)		240 (39)		283 (46)		619		

Entomological survey was carried out at homes of patients newly diagnosed with cutaneous or disseminated leishmaniasis. Sand fly data were stratified by sex and capture ecotype: intradomicile (Intra), peridomicile (Peri) and extradomicile (Extra)

^a^Relative abundance

In the 12 living sites of CL patients, 315 phlebotomine sand flies (51%) of 18 different species were collected (Additional file 1: Table S1), predominantly *Ny. whitmani* (*n* = 162; 51.4%), *Ny. intermedia* (*n* = 33; 10.5%), *Th. viannamartinsi* (*n* = 21; 6.7%), *Ev. bahiensis*, *Migonemyia migonei* (*n* = 18; 5.7%) and *Pintomyia fischeri* (*n* = 14; 4.4%). In the ten homes of patients with DL, 304 phlebotomine sand flies (49%) of 12 different species were collected, predominantly *Ny. whitmani* (*n* = 223; 73.4%), *Ny. intermedia* (*n* = 24; 7.9%), *Ev. bahiensis* (*n* = 21; 6.9%), *Mi. schreiberi* (*n* = 11; 3.6%), *Pi. fischeri* (*n* = 10; 3.3%), *Th. viannamartinsi* (*n* = 07; 2.3%) and *Mg. migonei* (*n* = 03; 1%) (Additional file: Table S1).

The Shannon diversity index (H’) was used to evaluate species diversity among the compared ecotypes and clinical form of ATL index cases. No significant difference was detected between the diversity of phlebotomine species in the residences of CL (H’ = 1.84) and DL (H’ = 1.07) patients (Mann-Whitney *U* test, *U* = 21.00, *Z* = − 1.44, *P* = 0.167). Although the effect of ecotype on diversity (H’*)* was not detected (Kruskal-Wallis H test, H = 5.301, df = 2, *P* = 0.071), extradomiciles presented significantly greater diversity of phlebotomine species (H’ = 1.71) than intradomiciles did (H’ = 0.92) in the residences surveyed (Mann-Whitney *U* test, *U* = 8.00, *Z* = − 2.07, *P* = 0.043).

Considering domiciliation as the sum of the data for intradomiciles and peridomiciles in our analyses, endophilic behavior could be observed in 54% of the phlebotomine sand flies collected, and there was no significant difference for an exophilic behavior, defined as its extradomicile abundance (Mann-Whitney *U* test, *U* = 14.50, *Z* = − 0.56, *P* = 0.589). Fifty-two and 56% of the sand flies from CL and DL residences, respectively, were collected from intradomiciliary and peridomiciliary ecotypes (Table 2), and no significant differences were detected between clinical forms of ATL index cases (Mann-Whitney *U* test, *U* = 13.50, *Z* = − 0.72, *P* = 0.485).

**Table 2:** Abundance and endophilic/exophilic behavior of the most frequent phlebotomine sand fly species collected between May 2018 and June 2019 in the Cacao Region, Bahia, Brazil

Species	CL		DL		Total^a^	
	Endophilic	Exophilic	Endophilic	Exophilic	Endophilic	Exophilic
*Nyssomyia whitmani*	94	68	130	93	224 (58%)	161 (42%)
*Nyssomyia intermedia*	18	15	23	01	41 (72%)	16 (28%)
*Evandromyia bahiensis*	07	11	08	13	15 (38%)	24 (62%)
*Trichophoromyia viannamartinsi*	11	10	01	06	12 (43%)	16 (57%)
*Pintomyia fischeri*	04	10	02	08	6 (25%)	18 (75%)
*Migonemyia migonei*	13	05	02	01	15 (71%)	6 (29%)
All species collected^a^	165 (52%)	150 (48%)	171 (56%)	133 (54%)	336 (54%)	283 (46%)

Entomological survey was carried out at homes of patients newly diagnosed with cutaneous leishmaniasis (CL) or disseminated leishmaniasis (DL)

^a^Relative abundance

The data in Table 2 show that, among the most abundant species, intradomiciles and peridomiciles concentrated 72% of *Ny. intermedia* and 71% of *Mg*. *migonei*, indicating high endophilic behavior, but there was no significant difference for an exophilic behavior (Mann-Whitney *U* test, *U* = 7.50, *Z* = − 1.08, *P* = 0.310 and Mann-Whitney *U* test, *U* = 4.50, *Z* = − 1.74, *P* = 0.095, respectively). However, *Mg*. *migonei* presented a significantly greater abundance in the peridomiciles than in the intradomiciles (Mann-Whitney *U* test, *U* = 2.00, *Z* = − 2.29, *P* = 0.032).

Exophilic behavior was observed for *Ev. bahiensis* and *Pi. fischeri*, with 62 and 75% of specimens collected from the extradomiciles (Table 2), without a statistically significant difference for endophilic behavior (Mann-Whitney *U* test, *U* = 7.50, *Z* = − 0.15, *P* = 0.886; Mann-Whitney *U* test, *U* = 10.00, *Z* = − 0.54, *P* = 0.690, respectively). Nevertheless, we detected statistically significant differences between the abundances of *Pi. fischeri* in the intradomiciles and extradomiciles (Mann-Whitney *U* test, *U* = 8.00, *Z* = − 2.35, *P* = 0.038) and between the abundances of *Ev. bahiensis* in the intradomiciles and peridomiciles surveyed (Mann-Whitney *U* test, *U* < 0.01, *Z* = − 2.46, *P* = 0.029).

*Nyssomyia whitmani* and *Th. viannamartinsi* were found in all ecotypes of the CL and DL residences, with endophilic behavior of 58% for the former and 43% for the latter sand fly species (Table 2). Among DL residences, *Ny. intermedia* presented an endophilic behavior of 96% and *Th. viannamartinsi* an exophilic behavior of 14%. However, no statistical significance could be detected for the differences between endophily and exophily for these two sand fly species (Mann-Whitney *U* test, *U* = 6.00, *Z* = − 1.54, *P* = 0.222 and Mann-Whitney *U* test, *U* = 5.50, *Z* = − 0.83, *P* = 0.486, respectively).

The natural infection rates of *L.* (*V.*) *braziliensis* were tested for 94% of the female sand flies collected. The specimens included in this analysis were pooled according to clinical form of ATL index cases, Phlebotominae species, and month and ecotype of sand fly capture. Among the 97 sand flies’ DNA pools, seven tested positive for *L.* (*V.*) *braziliensis*, three of which included *Ny. whitmani*, two of *Th. viannamartinsi*, and one of *Ps. hirsutus hirsutus* and *Ty. longispina*. The global MIR was 2.2%, whereas the stratification per species was 1.9% for *Ny. whitmani*, 10% for *Th. viannamartinsi*, 33% for *Psychodopygus hirsutus hirsutus* and 50% for *Trichopygomyia longispina* (Table 3). Notably, the qPCR technique was sensitive for detecting natural infection in samples with only one specimen of *Th. viannamartinsi* and *Ty. longispina*.

**Table 3:** Minimum infection rate (MIR) of phlebotomine sand fly species with *Leishmania* (*V*.) *braziliensis*

Species	♀ analyzed	Total of pools	Positive pools	MIR (%)
*Nyssomyia whitmani*	155	26	3	1.9
*Trichophoromyia viannamartinsi*	20	10	2	10
*Psychodopygus hirsutus hirsutus*	3	1	1	33
*Trichopygomyia longispina*	2	2	1	50
Others	144	58	–	–
Total	324	97	7	2.2

Entomological survey was carried out at homes of patients newly diagnosed with cutaneous leishmaniasis (CL) or disseminated leishmaniasis (DL) between May 2018 and June 2019 in the Cacao Region, Bahia, Brazil

All phlebotomine sand flies naturally infected with *L.* (*V.*) *braziliensis* were obtained during a single capture in one CL and one DL residence in the municipality of Taperoá in July 2018. We calculated MIR of 7.8% for this specific incursion, and 50% of the pools tested positive for *L.* (*V.*) *braziliensis*. The positive pools included four of the seven species found during that outbreak of ATL. Stratification per domestic environment displayed MIRs of 6.7% for domiciliation, 10.7% for peridomiciles and 8.9% for extradomiciles (Table 4).

**Table 4:** Minimum infection rate (MIR) of phlebotomine sand fly species with *Leishmania* (*V*.) *braziliensis*, stratified by capture ecotype and number of positive pools

Clinical form	Ecotype MIR (%)				Positive *pools*	Negative *pools*	Species
	Domiciliation^a^	Peridomicile	Extradomicile	Total			
CL	3.8	7.1	10.5	6.7	3	6	*Nyssomyia whitmani* and *Psychodopygus hirsutus hirsutus*
DL	10.5	14.3	7.7	8.9	4	2	*Nyssomyia whitmani*, *Trichophoromyia viannamartinsi* and* Trichopygomyia longispina*
Total	6.7	10.7	8.9	7.8	7	8	–

Results of a single entomological survey carried out in July 2018 at homes of patients newly diagnosed with cutaneous leishmaniasis (CL) or disseminated leishmaniasis (DL) in the municipality of Taperoá, Bahia, Brazil. ^a^Domiciliation = intra + peridomicile

We compared the CL and DL living sites, and the MIR in the CL residence was 6.7%, which was the lowest in the domestic environment (3.8%), whereas for the DL residence, it was 8.9%, with the peridomiciles being the highest at 14.3% (Table 4). When we evaluated differences between positive pools of CL and DL living sites, no statistical significance could be detected in this specific incursion (*χ2* = *1.60, df* = *1, P* = *0.31).*

## Discussion

The current findings suggest that the ecoepidemiological profile of ATL in the major spatial circuit of the disease in Bahia, northeast Brazil, can be classified as ‘modified silvatic’ [18], with an enzootic cycle involving *L.* (*V.*) *braziliensis* and wild mammals, occurring in a forested environment where transmission of the parasite among hosts involves the exophilic sand fly species *Pi. fischeri*, *Ps. hirsutus hirsutus*, *Th. viannamartinsi* and *Ty. longispina*. The enzootic cycle would then spill over to zoonotic peridomiciliary transmission of *L.* (*V.*) *braziliensis* among human beings, domestic and synanthropic mammals, involving mainly the endophilic sand fly species *Ny. intermedia*, *Ny. whitmani* and possibly *Mg. migonei*.

The replacement of the primary forest by plantations, mainly of cacao crops, commonly grown in forested areas, is one factor that could help explain the high *L.* (*V.*) *braziliensis* transmission to humans in the Cacao Region. Several aspects found in the current study seem to favor the domiciliary transmission of ATL characteristic of the ‘modified silvatic’ pattern [18] in that region: (i) the high abundance of male sand flies in our collection indicates the presence of active phlebotomine sand flies breeding around the households surveyed; (ii) extradomiciles presented significantly greater species diversity and abundance of phlebotomine sand flies than intradomiciles did; (iii) some synanthropic species, such as *Ny. intermedia* and *Ny. whitmani*, presented higher densities in animal shelters close to human habitation than inside the forest.

Environmental modifications from anthropic action may have contributed to the domiciliation of *Ny. intermedia* and *Ny. whitmani* [9], present in *L.* (*V.*) *braziliensis*-endemic areas in southern Bahia [30–37]. ATL ecoepidemiology has been extensively studied in that region, with the earliest report of *L.* (*V.*) *braziliensis* infecting *Ny. whitmani* dating from the 1980s [36]. In endemic municipalities in the Cacao Region, *Ny. whitmani* has been collected in proximity to human beings within crops maintained by local dwellers, indicating the anthropophilic tendency of this sand fly vector [30, 32, 33].

*Nyssomyia intermedia*, *Ny. whitmani*, *Mg. migonei* and *Th. viannamartinsi* presented endophilic behaviors in this study, whereas exophilic behaviors could be observed for *Ev. bahiensis* and *Pi. fischeri*. Nevertheless, no statistical significance could be detected for the differences between endophily and exophily for all phlebotomine sand fly species. This may be because the environmental conditions have not completely changed in affected areas, maintaining a continuous interchange between domestic and sylvatic transmission cycles of *L.* (*V.*) *braziliensis* [38].

In the present study, we compared the phlebotomine fauna between geographically matched households presenting index cases of distinct ATL forms (i.e. CL or DL). No significant differences could be observed for MIRs, abundance, richness, diversity and endophily/exophily of the different sand fly species collected at the matched households. This suggests that phlebotomine sand flies may not play a major role in conditioning ATL manifestations in infected individuals, for example by transmitting *L.* (*V.*) *braziliensis* genotypes associated with a greater risk of CL or DL [8, 39]. Notably, *Ny. whitmani* was the most abundant and the only sand fly species naturally infected with *L.* (*V.*) *braziliensis* found in both CL and DL residences.

We detected phlebotomine sand flies naturally infected with *L.* (*V.*) *braziliensis* during a single capture that occurred in Taperoá in July 2018. The absence of female sand flies naturally infected in more than one incursion during the current study did not allow us to further evaluate differences between the MIRs of the CL/DL residences.

Miranda et al. conducted an entomological survey in the same region in southern Bahia during the 1990s [37]. They collected 4000 female sand flies in the domestic and peridomestic environments of local residences, 93% of which consisted of *Ny. whitmani*. These females were divided into 335 pools, each one with 10 to 20 specimens for the analysis of natural infection by *L*. (*V*.) *braziliensis* via PCR/dotblot. Approximately 9% of the pools tested positive for the parasite, with a total MIR of 0.4%. That MIR was substantially lower than what we found in the current study for *Ny. whitmani* (1.9%). Furthermore, 83.3% of the *L.* (*V.*) *braziliensis*-positive pools observed by those authors were concentrated within a single region of the ATL study site, resulting in a higher value for the regionalized MIR (1.5%). This difference between total and regionalized MIRs somewhat parallels the total MIR of 2.2% and regionalized Taperoá-specific MIR of 7.8% that we observed in the current study.

All phlebotomine sand fly species collected in this study had been previously recorded in Bahia [15]. The Brazilian Ministry of Health classifies ATL-implicated sand fly species as proven or putative vectors of *Leishmania* spp. [25]. *Evandromyia* (*Barretomyia*) *bahiensis* was the third most abundant species in our collection but has not been classified as an ATL vector by local public authorities. Specimens of the *Barretomyia* subgenus (Martins & Silva, 1968) have been found naturally infected with *L.* (*V.*) *braziliensis* in southern Bahia [36].

*Migonemyia migonei* and *Pi. fischeri* have been recognized as potential ATL vectors for peridomiciliary ecotypes [9]. These species have been previously found in entomological surveys carried out in southern Bahia [30–34]. *Migonemyia migonei* is divided into two subpopulations in Bahia: central, in a region dominated by savannah, and coastal, spread within the Atlantic rainforest biome. *Pintomyia fischeri* is more common in the Atlantic rainforest [15], which includes the Cacao Region.

*Pintomyia fischeri*, *Mg. migonei* and *Ps. hirsutus hirsutus* in the present study may act as accessory vectors, probably helping maintain the enzootic cycle of *Leishmania* spp. in secondary vegetation but also being capable of feeding on humans and domestic animals [40]. These sand fly species are primarily sylvatic but have probably adapted to peridomestic and domestic habitats because of deforestation in the Cacao Region. *Migonemyia migonei* presented endophilic behavior in the current survey, whereas *Pi. fischeri* and *Ps. hirsutus hirsutus* presented exophilic behaviors.

*Migonemyia migonei* has been found naturally infected with *L.* (*V.*) *braziliensis* in southeast Brazil [41]. The abundance of *Pi. fischeri* with high anthropophilic levels in the rural outskirts of ​​Ilhéus, southern Bahia, suggests its role as a secondary ATL vector in that region [31]. This species has also been found naturally infected with *Leishmania (Viannia)* spp. in other Brazilian regions [42, 43].

*Psychodopygus hirsutus hirsutus* presented a MIR of 33% in the current study. This species occurs mainly in the Atlantic rainforest in southeastern Bahia [15] and has also been found infected with *Leishmania* (*Viannia*) spp. in Rio de Janeiro [9]. Another species of this genus, *Psychodopygus ayrozai* (Barretto & Coutinho, 1940), has been found at high density, presenting anthropophilic behavior in cacao crop areas within the Ubaíra municipality in the Jiquiriçá Valley, southeastern Bahia [34]. Still another species, *Ps. davisi*, has ATL epidemiological importance in the Amazon [44]. It has been found in abundance in ATL-endemic areas, with MIRs for *L.* (*V.*) *braziliensis* of 1.1% in Acre [45] and 0.05% in Rondônia [46].

There are reports of natural infection of *Ty. longispina* with *L.* (*V.*) *braziliensis* in the Atlantic rainforest region of Pernambuco, Northeastern Brazil [47]. This species presented a MIR of 50% in the current study, which is much higher than the 2% observed in Pernambuco. However, our figure is imprecise because only two female sand flies of this species could be collected, resulting in a single positive pool. In Pernambuco, 498 female sand flies were collected from 10 positive pools [47]. This species is frequently found in armadillo burrows, which suggests its participation in the enzootic cycle of *Leishmania* spp. [48]. There is still no evidence that *Ty. longispina* transmits the parasite to humans.

To our knowledge, natural *Th. viannamartinsi* infection with *L.* (*V.*) *braziliensis* has not yet been reported in the literature. This species was described in the 1970s and was initially considered in synonymy with *Trichophoromyia brachipyga* Mangabeira, 1942 [49], which presents minimal criteria to be included in the list of suspected leishmaniasis vectors [50]. The epidemiological relevance of *Trichophoromyia* spp. is suggested by high abundance in ATL endemic foci in the Amazonian states of Acre, Amapá and Rondônia, where it has been found naturally infected with *L.* (*V.*) *braziliensis* [45, 46, 51].

There has been a marked increase in the number of proven and putative leishmaniasis vectors in the New World. This has been supported mainly by highly sensitive PCR-based techniques capable of detecting *Leishmania* spp. genes within DNA extracted from individual or pooled phlebotomine sand flies [23]. However, the classification of sand fly species as suspected vectors exclusively by molecular methods may be misleading. Proper classification should involve assessments of parasite DNA present in the phlebotomine nucleic acid extract, estimation of the parasitic load and confirmation that infective forms of *Leishmania* spp. can indeed be recovered from the implicated sand fly samples [50].

Xenodiagnosis should also be mandatory for demonstrating the vectorial capacity of phlebotomine sand fly species. Our finding of *Th. viannamartinsi* infection with *L.* (*V.*) *braziliensis* reinforces the relevance of testing these criteria in *Trichophoromyia* spp. New species of this genus have been described, and its taxonomic identification based on the morphology of females is particularly difficult, becoming a challenge in instances of geographic overlap among different sand fly species included this genus [50].

There was a time lag between ATL index case infections with *L.* (*V.*) *braziliensis* and the collection of phlebotomine sand flies at their residences in the current study, caused by (i) ATL incubation time in humans, (ii) the patient's visit to the leishmaniasis clinic, (iii) the molecular diagnosis of patient infections with *L.* (*V.*) *braziliensis* by real-time PCR and (iv) the process of recruiting CL and DL index case residences where phlebotomine sand fly traps were set up.

We have previously shown that the *L.* (*V.*) *braziliensis* endemics in the study region consist of concurring and successive outbreaks of human cases of ATL [19, 20]. The finding of phlebotomine sand flies infected with *L.* (*V.*) *braziliensis* in only one of nine incursions during this entomological survey that may have been caused by the combination of a greater abundance of phlebotomine sand flies around the enrolled CL and DL residences at Taperoá in July 2018 and the self-reports of the recruited index cases to the leishmaniasis clinic during the early stages of this incoming outbreak.

## Conclusions

Our findings suggest that areas experiencing outbreaks of ATL in the Cacao Region present high proportions of infected phlebotomine sand flies involving a variety of species, some not usually considered involved in the *L.* (*V.*) *braziliensis* transmission cycle, such as *Th. viannamartinsi*. Phlebotomine sand flies do not seem to play a major role in conditioning ATL clinical manifestations in infected individuals, for example by transmitting *L.* (*V.*) *braziliensis* genotypes associated with a greater risk of CL or DL. Entomological surveillance studies are necessary to further reveal species of phlebotomine sand flies with *Leishmania* spp. vectorial competence in ATL-endemic regions of Northeast Brazil, such as the Cacao Region in southern Bahia.

## Supplementary Information

Supplementary Material 1: Table S1. Phlebotomine sand fly species collected between May 2018 and June 2019 in the Cacao Region, Bahia, Brazil. Entomological survey was carried out at homes of patients newly diagnosed with cutaneous leishmaniasisor disseminated leishmaniasis

## Data Availability

No datasets were generated or analyzed during the current study.
