# Supplementary material for: Diversity and natural infection of phlebotomine sand flies (Diptera, Psychodidae) in an endemic area of American tegumentary leishmaniasis in southeastern Bahia, Brazil
Source: Parasit Vectors. 2025 Feb 26;18:79. doi: 10.1186/s13071-025-06717-y (PMC11866633; doi:10.1186/s13071-025-06717-y)
Supplement: Supplementary file 1 — Supplementary Material 1: Table S1. Phlebotomine sand fly species collected between May 2018 and June 2019 in the Cacao Region, Bahia, Brazil. Entomological survey was carried out at homes of patients newly diagnosed with cutaneous leishmaniasisor disseminated leishmaniasis [file 13071_2025_6717_MOESM1_ESM.docx]

**Table S1. Phlebotomine sand fly species collected between May 2018 and June 2019 in the Cacao Region, Bahia, Brazil.** Entomological survey was carried out at homes of patients newly diagnosed with Cutaneous Leishmaniasis (CL) or Disseminated Leishmaniasis (DL).

| Species | May 18 | | June 18 | | July 18 | | Aug 18 | | Octob 18 | | Febru 19 | | | March 19 | | | April 19 | | June 19 | | Total | | Total^a^ |
| --- | --- | --- | --- | --- | --- | --- | --- | --- | --- | --- | --- | --- | --- | --- | --- | --- | --- | --- | --- | --- | --- | --- | --- |
|  | CL | DL | CL | DL | CL | DL | CL | DL | CL | DL | CL | DL | | CL | DL | | CL | DL | CL | DL | CL | DL |  |
| *Ny. whitmani* | 02 | - | 30 | 23 | 69 | 104 | 05 | 03 | 02 | 06 | 41 | | 01 | - | - | | 12 | 72 | 01 | 14 | 162 | 223 | 385 (62.2) |
| *Ny. intermedia* | - | - | 23 | 19 | - | - | - | 03 | 01 | - | 04 | | 02 | 05 | - | | - | - | - | - | 33 | 24 | 57 (9.2) |
| *Ev. bahiensis* | - | - | - | - | - | - | - | - | - | - | - | | 15 | 05 | 01 | | 01 | - | 12 | 05 | 18 | 21 | 39 (6.3) |
| *Th. viannamartinsi* | 04 | - | 01 | - | 05 | 02 | 11 | 05 | - | - | - | | - | - | - | | - | - | - | - | 21 | 07 | 28 (4.5) |
| *Pi. fischeri* | 02 | - | - | - | 01 | - | 02 | - | 02 | 01 | 04 | | 04 | - | 02 | | 02 | 01 | 01 | 02 | 14 | 10 | 24 (3.9) |
| *Mg. migonei* | - | - | 02 | - | - | - | - | - | 05 | 01 | 01 | 02 | | 08 | | - | 02 | - | - | - | 18 | 03 | 21 (3.4) |
| *Mi. schreiberi* | - | - | - | - | - | - | - | - | - | - | 02 | 02 | | - | | - | 03 | 08 | - | 01 | 05 | 11 | 16 (2.6) |
| *Ev. tupynambai* | 02 | - | 02 | - | 01 | - | - | - | - | - | 06 | - | | - | | - | - | - | - | - | 11 | - | 11 (1.8) |
| *Pa. aragaoi* | - | - | 10 | - | - | - | - | - | - | - | - | - | | - | | - | - | - | - | - | 10 | - | 10 (1.6) |
| *Pr. choti* | - | - | - | - | - | - | - | - | - | - | 09 | - | | - | | - | - | - | - | - | 09 | - | 09 (1.5) |
| *Pa. bigeniculata* | - | - | - | - | - | - | 01 | - | 02 | - | - | - | | - | | - | - | - | - | - | 03 | - | 03 (0.5) |
| *Pa. pascalei* | - | - | 02 | - | 01 | - | - | - | - | - | - | - | | - | | - | - | - | - | - | 03 | - | 03 (0.5) |
| *Ps. hirsutus hirsutus* | - | - | - | - | 03 | - | - | - | - | - | - | - | | - | | - | - | - | - | - | 03 | - | 03 (0.5) |
| *Mi. oswaldoi* | - | - | 01 | - | - | - | - | - | - | - | - | 01 | | - | | - | - | - | - | - | 01 | 01 | 02 (0.3) |
| *Pi. serrana* | - | - | - | - | - | - | - | - | - | - | - | - | | 01 | | 01 | - | - | - | - | 01 | 01 | 02 (0.3) |
| *Ty. longispina* | - | - | - | - | 01 | 01 | - | - | - | - | - | - | | - | | - | - | - | - | - | 01 | 01 | 02 (0.3) |
| *Br. cunhai* | - | - | - | - | - | - | - | - | - | - | - | - | | - | | - | - | - | - | 01 | - | 01 | 01 (0.2) |
| *Mi. capixaba* | - | - | 01 | - | - | - | - | - | - | - | - | - | | - | | - | - | - | - | - | 01 | - | 01 (0.2) |
| *Pa. barretoi barretoi* | - | - | - | - | - | - | - | 01 | - | - | - | - | | - | | - | - | - | - | - | - | 01 | 01 (0.2) |
| *Pa. lanei* | - | - | 01 | - | - | - | - | - | - | - | - | - | | - | | - | - | - | - | - | 01 | - | 01 (0.2) |
| Total | 10 | - | 73 | 42 | 81 | 107 | 19 | 12 | 12 | 08 | 67 | 27 | | 19 | | 04 | 20 | 81 | 14 | 23 | 315 | 304 | 619 (100) |
|  | 10 | | 115 | | 188 | | 31 | | 20 | | 94 | | | 23 | | | 101 | | 37 | |  |  |  |

^a^Relative Abundance
